# Quercetin induces pannexin 1 expression via an alternative transcript with a translationally active 5′ leader in rhabdomyosarcoma

**DOI:** 10.1038/s41389-022-00384-9

**Published:** 2022-02-22

**Authors:** Xiao Xiang, Huy-Dung Hoang, Victoria H. Gilchrist, Stéphanie Langlois, Tommy Alain, Kyle N. Cowan

**Affiliations:** 1 Molecular Biomedicine Program, Children’s Hospital of Eastern Ontario, Ottawa, ON Canada; 2 Department of Cellular and Molecular Medicine, University of Ottawa, Ottawa, ON Canada; 3 Department of Biochemistry, Microbiology and Immunology, University of Ottawa, Ottawa, ON Canada; 4 Department of Surgery, Children’s Hospital of Eastern Ontario, University of Ottawa, Ottawa, ON Canada

**Keywords:** Paediatric cancer, Cancer genetics

## Abstract

Rhabdomyosarcoma (RMS) is a deadly cancer of skeletal muscle origin. Pannexin 1 (PANX1) is down-regulated in RMS and increasing its levels drastically inhibits RMS progression. PANX1 upregulation thus represents a prospective new treatment strategy for this malignancy. However, the mechanisms regulating PANX1 expression, in RMS and other contexts, remain largely unknown. Here we show that both RMS and normal skeletal muscle express a comparable amount of *PANX1* mRNAs, but surprisingly the canonical 5′ untranslated region (5′ UTR) or 5′ leader of the transcript is completely lost in RMS. We uncover that quercetin, a natural plant flavonoid, increases PANX1 protein levels in RMS by inducing re-expression of a 5′ leader-containing *PANX1* transcript variant that is efficiently translated. This particular *PANX1* mRNA variant is also present in differentiated human skeletal muscle myoblasts (HSMM) that highly express PANX1. Mechanistically, abolishing ETV4 transcription factor binding sites in the *PANX1* promoter significantly reduced the luciferase reporter activities and *PANX1* 5′ UTR levels, and both quercetin treatment in RMS cells and induction of differentiation in HSMM enriched the binding of ETV4 to its consensus element in the *PANX1* promoter. Notably, quercetin treatment promoted RMS differentiation in a PANX1-dependent manner. Further showing its therapeutic potential, quercetin treatment prevented RMS in vitro tumor formation while inducing complete regression of established spheroids. Collectively, our results demonstrate the tumor-suppressive effects of quercetin in RMS and present a hitherto undescribed mechanism of PANX1 regulation via ETV4-mediated transcription of a translationally functional 5′ leader-containing *PANX1* mRNA.

## Introduction

Pannexin 1 (PANX1; known as Panx1 in rodents) is a transmembrane glycoprotein forming single membrane channels that are considered major conduits for ATP release [1–5]. We have previously shown that PANX1 levels are highly upregulated during human skeletal muscle myoblast (HSMM) differentiation [6], as well as during murine skeletal muscle development and regeneration [7]. PANX1 over-expression in undifferentiated HSMM promoted their differentiation while PANX1 channel blockade inhibited this process [6]. After establishing PANX1 as an important regulator of myogenesis [8], we investigated its role in rhabdomyosarcoma (RMS), a neoplasm thought to arise from muscle progenitors due to impaired differentiation [9–11]. PANX1 levels are down-regulated in RMS tissue specimens and patient-derived cell lines. Notably, ectopic expression of PANX1 in RMS cells dramatically suppressed their malignant properties in vitro and in vivo [12]. Our study of the PANX1 transcriptome and interactome in RMS further implicated PANX1 in the regulation of genes involved in various key cellular processes, such as migration and apoptosis, and uncovered its tumor-inhibitory interaction with the neuroblast differentiation-associated protein AHNAK, respectively [13].

RMS is the most common soft tissue sarcoma in children and adolescents with two major histological subtypes, termed embryonal RMS (eRMS) and alveolar RMS (aRMS) [14]. eRMS has a more favorable prognosis whereas aRMS is more aggressive and often leads to a poor clinical outcome [14–17]. Despite aggressive treatment modalities, the overall survival rate of patients with metastatic or relapsed RMS has remained below 30%, underlining the urgency for better clinical management [16, 18]. Our findings strongly suggest that upregulating PANX1 is a promising therapeutic strategy for RMS [12]. As PANX1 levels are low in RMS compared to skeletal muscle, understanding the regulation or deregulation of *PANX1* transcriptional and translational control in RMS may be of therapeutic benefit.

PANX1/Panx1 levels are dynamically regulated during development and in pathologic states. In murine cortex, cerebellum, and retina, Panx1 levels peaked at embryonic day 18 and dramatically decreased upon birth and continued to decline into adulthood [19]. This decline in Panx1 levels was also reported in the rat brain [20] and during the differentiation of neural crest-like cells in vitro [19]. In contrast, Panx1 levels increased in primary adipose-derived stromal cells upon induction into an adipogenic lineage [21]. Under pathological conditions, Panx1 levels were down-regulated in C6 glioma cells as compared to normal astrocytes [22]. By contrast, upregulation of PANX1/Panx1 levels was positively associated with human and murine melanoma progression [23, 24]. This dynamic variation of PANX1 levels across different tissue types or pathological conditions suggests tissue- or disease-dependent transcriptional or translational regulation [25]. However, studies demonstrating the mechanisms by which this regulation occurs have not been reported. Thus far, *Panx1* transcriptional regulation has only been comprehensively studied in the rat epididymis, where *Panx1* was shown to have an evolutionarily conserved promoter located in CpG islands under the control of methylation and the transcription factors CREB and ETV4 [26].

Recently, a genome-wide drug screen in murine neuronal cells revealed quercetin, a naturally occurring flavonoid [27], as an upregulator of *Panx1* mRNA levels [28]. Thus providing a pharmacological agent that could facilitate the investigation into the molecular mechanism of *PANX1* regulation in RMS. In the current study, using Rh18 (eRMS) and Rh30 (aRMS) cell lines [29], we show that *PANX1* transcripts in RMS cells are devoid of their putative 5′ UTR (untranslated region) as compared to skeletal muscle tissue and differentiated HSMM, and that the presence of the *PANX1* 5′ UTR corresponds to its protein expression in RMS cells and HSMM. In RMS cells, quercetin upregulates PANX1 protein levels through the restoration of transcription of a translationally functional *PANX1* mRNA containing a 5′ leader. This mRNA was also detected in differentiated HSMM, which highly express PANX1. The transcription of this *PANX1* mRNA variant is dependent upon the binding of the transcription factor ETV4 to the *PANX1* promoter. Notably, quercetin treatment promoted RMS differentiation in a PANX1-dependent manner. Further showing its therapeutic potential, quercetin treatment prevented RMS in vitro tumor formation while inducing complete regression of established spheroids. Collectively, our results show for the first time the involvement of the *PANX1* 5′ UTR in regulating its mRNA translation and the tumor-suppressive effects of quercetin in RMS.

## Materials and methods

See “Supplemental Material and Methods” for detailed information on methods and antibodies. Primers are described in Supplemental Table S1.

### Cells, drug treatment, and transfection

Rh18 and Rh30 cell lines were from Dr. P. Houghton (St. Jude Children’s Hospital, Memphis, TN). Stable Rh18 and Rh30 cell lines were previously generated [12]. HSMM was from Lonza (Walkersville, MD). The HSMM was differentiated by switching to DMEM medium with 2% horse serum [6]. Quercetin (Sigma-Aldrich, St. Louis, MO) was used at 50 μM and 10 μM for Rh18 and Rh30 cells, respectively, for 24 h unless indicated otherwise. For experiments requiring longer treatment, the drug was refreshed in culture medium every 24 h. Transfections were performed with Lipofectamine 2000 Reagent (Thermo Scientific, Waltham, MA).

### Western blotting

Cell lysates were obtained and analyzed as previously described [30].

### RNA interference

Cells were transfected with Silencer® Select siRNA targeting *PANX1* or Silencer® Select Negative Control No. 1 (Life Technologies) for 72 h.

### RNA sequencing and data analysis

Total RNA was extracted from Rh30 cells and submitted to Princess Margaret Genomics Centre (Toronto, ON, Canada) for RNA-seq analysis as previously described [12].

### Polysome profiling

Polysome profiling was performed as previously described [31].

### RT-qPCR

Total RNA was extracted, DNase-treated, reverse transcribed into cDNA, and analyzed as described in “Supplemental Material and Methods”.

### 5′ Rapid Amplification of cDNA End (RACE)

Rh30 cells were treated with 10 µM quercetin or DMSO for 24 h, while HSMM were used at 0 (undifferentiated) or 48 h (differentiated) following serum starvation to induce differentiation. Total RNA was collected and DNase-treated. Reverse transcription of 5′ capped poly (A) RNA was performed with TELO^TM^ PRIME Full-Length cDNA Amplification V2 kit following manufacturer’s instructions.

### CAT translation reporter assay

CAT translation reporter assay was performed as previously described [32].

### PANX1 promoter cloning, plasmid construction, and site-directed mutagenesis

Total genomic DNA was extracted from HSMM. Two segments of *PANX1* promoter from positions -2697 to -1642 and −1616 to +38 as well as their deletion mutants from positions −926, −581, and −475 to +38 (relative to its ATG start codon) were PCR amplified, purified, and subcloned into the pGL3-Basic vector (Promega). The lack of the 26 bp between −1642 and −1616 in the *PANX1* promoter is due to challenges in cloning this GC-rich region. In silico prediction yielded no potential transcription factors in this short fragment.

Mutations at the CREB and ETV4 consensus sites on *PANX1* promoter (−581 to +38) in pGL3 vector were introduced using the Quick-Change Site-directed Mutagenesis kit (Agilent Technologies, Santa Clara, CA).

### Immunofluorescence microscopy

Rh30 cells on glass coverslips were treated with 10 µM quercetin or DMSO for 24 h. Immunofluorescent labeling was performed as previously described [12].

### Dual-luciferase reporter assay

Cells were transfected with pGL3-*PANX1* promoter constructs or a promoterless pGL3-Basic negative and pRL-TK (Promega) for 48 h and analyzed using Dual-Glo^®^ Luciferase Assay System (Promega). Cells were transfected in parallel with pGL3 constructs and pRL-TK for 48 h before total RNA extraction for qPCR analyses of *Firefly Luciferase* and *PANX1* 5′ UTR transcript levels.

### Chromatin Immunoprecipitation

Rh30 cells were treated with 10 µM quercetin or DMSO for 24 h and subjected to chromatin immunoprecipitation using the EZ-ChIP kit (Millipore, Billerica, MA).

### 3D spheroid assay

3D spheroid assays were performed as previously described [12].

### Statistics

Paired or unpaired two-tailed Student’s *t* tests, multiple Student’s *t* test with Holm–Sidak correction, and one-way or two-way analysis of variance (ANOVA) followed by Tukey’s post-hoc tests were used. Results are given as mean ± s.d. Results with *P* < 0.05 were considered significant. The number of times each experiment was performed is indicated in the figure legends with the individual data points displayed on the graphs.

## Results

### PANX1 5’ UTR and protein levels are positively correlated in RMS and HSMM

**Fig. 1:**
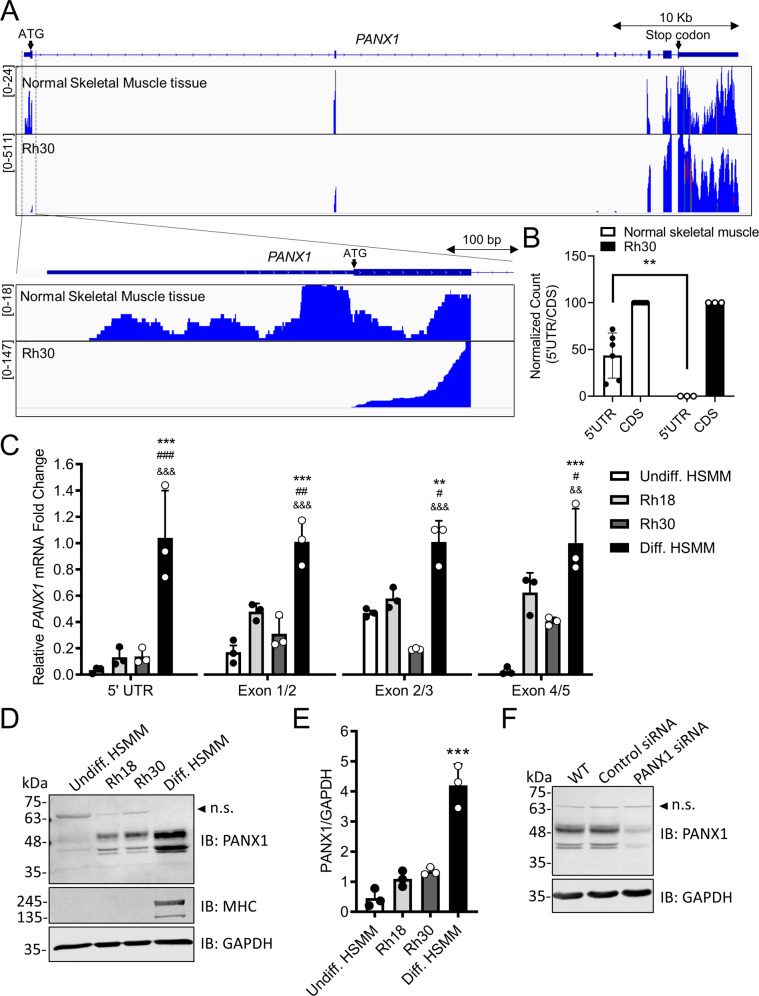
PANX1 5′ UTR expression correlates with its protein abundance. **A** RNA-seq reads mapped to the 5′ UTR and exon 1 regions of *PANX1* from representative RNA libraries prepared from three biological replicates of Rh30 (aRMS) cells and as compared to representative RNA libraries from six normal human skeletal muscle tissue samples retrieved from Ryan et al., *JCI Insight* 2018. The Y-axis shows RNA-seq read coverage. **B** Ratio of normalized counts of RNA-seq reads mapped to the 5′ UTR region over the CDS region of *PANX1*. Note that there is no read mapped to the 5′ UTR region of *PANX1* in Rh30 RNA samples. All normal skeletal muscle RNA-seq reads were retrieved from Ryan et al., *JCI Insight* 2018. Statistical analysis was performed using a two-way ANOVA with Tukey’s post-hoc test. **P < 0.01 between normal skeletal muscle and Rh30. **C** RT-qPCR of *PANX1* from Rh18 (eRMS) (*n* = 3) and Rh30 cells (n = 3) using primers specific to its 5′ UTR (−276 to +1 relative to the *PANX1* ATG start codon) and exon-exon junctions. Undifferentiated (*n* = 3) and differentiated (*n* = 3) human skeletal muscle myoblasts (HSMM) were used as controls. Results from each amplified region were normalized independently to their differentiated (Diff. HSMM) HSMM controls in the respective *PANX1* mRNA regions. **P < 0.01 and ***P < 0.001 compared to Undiff. HSMM; ^#^*P* < 0.05,^##^*P* < 0.01 and ^###^*P* < 0.001 compared to Rh18; ^&&^*P* < 0.01 and ^&&&^*P* < 0.001 compared to Rh30 (*n* = 3). **D** Representative Western blots and their quantification **E** showing levels of myosin heavy chain (MHC), a marker for terminal myogenic differentiation of HSMM, and PANX1 (*n* = 3). GAPDH was used as loading control. Arrowhead indicates a non-specific immunoreactive band (n.s.), which was further confirmed by (**F**) Western blotting analysis (*n* = 3) of Rh30 cells 72 h post-siRNA-mediated knockdown of PANX1. Both wildtype Rh30 cells (WT) and Rh30 cells transfected with a scrambled siRNA sequence (Control siRNA) were used as controls. GAPDH was used as loading control. Arrowhead highlights the non-specific immunoreactive band (n.s.) also seen in (**D**). ***P < 0.001 compared to Undiff. HSMM, Rh18, and Rh30. Results are expressed as mean ± s.d. Statistical analysis was performed using one-way ANOVA with Tukey’s post-hoc tests in (**C**) and (**E**).

Unexpectedly, the RNA-sequencing (RNA-seq) result from Rh30 (aRMS) cells revealed an abundance of endogenous *PANX1* transcripts which mapped to all five exons with the exception of the 5′ UTR region (Fig. 1A). However, RNA-seq data on adult skeletal muscle tissue specimens deposited in the Gene Expression Omnibus (GEO) database showed a clear mapping of *PANX1* transcripts onto its 5′ UTR region (Fig. 1A). When normalized to the total RNA-seq reads of the *PANX1* coding sequence (CDS), the 5′ UTR reads in normal muscle were comparable to the CDS reads, while no 5′ UTR read was detected in Rh30 RNA samples, suggesting that *PANX1* mRNAs in Rh30 cells do not contain a 5′ leader region (Fig. 1B). qPCR analyses corroborated the previous RNA-seq results by showing that the difference in *PANX1* transcript levels between Rh18 (eRMS), or Rh30 cells, and differentiated HSMM was the most dramatic in the 5′ UTR region, and this difference gradually diminished from exon 1 to 5 (Fig. 1C). Western blots in parallel showed higher PANX1 protein levels in differentiated HSMM as compared to that of Rh18 and Rh30 cells as well as its undifferentiated counterparts (Fig. 1D, E). Antibody specificity was confirmed using siRNA targeting PANX1 (PANX1 is detected between ~39.5 to ~49.9 kDa due to post-translational modifications [33]) (Fig. 1F). Taken together, our data show that as compared to HSMM, the *PANX1* transcripts expressed in RMS cells are devoid of a 5′ leader region and this correlates with protein expression.

### Quercetin enhances *PANX1* mRNA translation in RMS cells

**Fig. 2:**
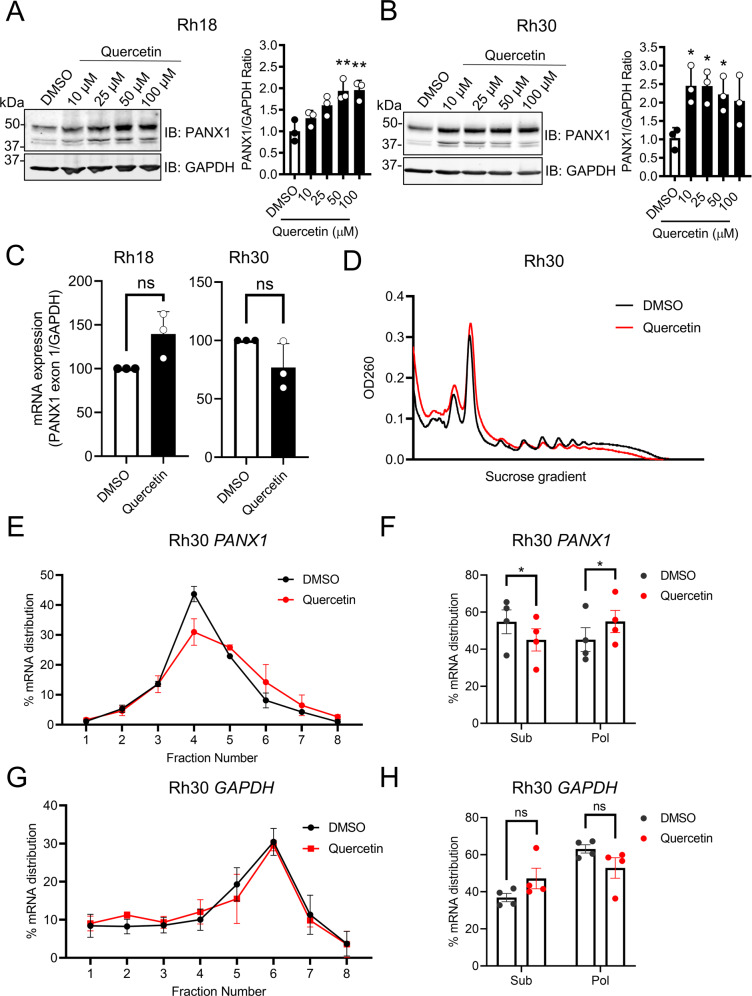
Quercetin upregulates the translation of *PANX1* mRNA in RMS cells. Rh18 (eRMS) and Rh30 (aRMS) cells were treated with increasing dosages of quercetin or its vehicle control (DMSO) for 24 h. DMSO concentration corresponded to the highest dosage of quercetin. Representative Western blots and quantification for PANX1 in Rh18 (*n* = 3) (**A**) and Rh30 (*n* = 3) (**B**) cells are shown. Statistical analysis was performed using a one-way ANOVA with Tukey’s post-hoc test. *P < 0.05 and **P < 0.01 compared to DMSO. GAPDH was used as loading control. Rh18 and Rh30 cells were treated with 50 μM and 10 μM, respectively, for 24 h and subjected to RT-qPCR or polysome fractionation. **C** RT-qPCR analysis of *PANX1* transcript levels in Rh18 (*n* = 3) and Rh30 (*n* = 3) using primers specific to its exon 1 region. Results are relative to *GAPDH* transcript levels. ns: not significant. qPCR program: 95 °C for 3 min followed by 40 cycles of 95 °C for 15 s, 60 °C for 30 s. Statistical analysis was performed using a paired Student’s *t* test. ns not significant. **D** Representative polysome traces of Rh30 cells treated with quercetin or DMSO. RT-qPCR analysis (*n* = 4) showing (**E**) distribution of *PANX1* mRNA levels in polysome fractions and (**F**) their proportions in subpolysomes (fractions 1–4) and polysomes (fractions 5–8) fractions from DMSO- or quercetin-treated Rh30 cells. Results are expressed as the percentage of total *PANX1* mRNA. Statistical analysis was performed using a paired Student’s *t* test. *P < 0.05. RT-qPCR analysis (*n* = 4) showing (**G**) distribution of *GAPDH* mRNA levels in polysome fractions and (**H**) their proportions in combined subpolysomes (fractions 1–4) (translationally inactive) and polysomes (fractions 5–8) (translationally active) fractions from the DMSO- or quercetin-treated Rh30 cells in (**E**) and (**F**). Results are expressed as the percentage of total *GAPDH* mRNA. Statistical analysis was performed using a paired Student’s *t* test. ns not significant.

Quercetin treatment induced a dosage-dependent increase in PANX1 protein levels in Rh18 cells in which 50 µM of quercetin induced a statistically significant and maximal increase of PANX1 (Fig. 2A). In Rh30 cells, 10 µM of quercetin was able to elicit maximal induction of PANX1 expression (Fig. 2B). Thus, 50 µM and 10 µM were chosen as quercetin concentrations for treating Rh18 and Rh30 cells in the downstream experiments, respectively. Remarkably, quercetin treatment (24 h) in both Rh18 and Rh30 cells did not lead to a significant increase in *PANX1* transcript levels (Fig. 2C) prompting us to investigate whether quercetin treatment could instead modulate *PANX1* mRNA translation. Using polysome fractionation [34], ribosome-associated mRNA from Rh30 cells with and without quercetin treatment (10 µM; 24 h) was resolved on a continuous sucrose gradient where polysome-bound mRNA sedimented according to their density, which increases with the number of bound ribosomes and correlates with their translation rate (Fig. 2D). While there was no discernable change in the global translation profiles (Fig. 2D), the distribution of *PANX1* transcripts with quercetin treatment shifted towards the polysome fractions 5–8 when analyzed by qPCR (Fig. 2E). Fractions were further grouped into translationally inactive subpolysomes (fractions 1–4) or translationally active polysomes (fractions 5–8). As expected, quercetin treatment (10 µM; 24 h) induced a significant increase of the proportion of *PANX1* transcripts in the polysome fraction and a corresponding decrease of those in the subpolysome fraction (Fig. 2F). In contrast, the distribution of *GAPDH* transcripts across polysome fractions (Fig. 2G) and when comparing their proportions between subpolysome and polysome fractions (Fig. 2H), both showed no change upon quercetin treatment (10 µM; 24 h). Collectively, our data indicate that quercetin induced PANX1 expression in Rh30 cells by enhancing its translation.

### Quercetin-induced transcription of a skeletal muscle relevant translationally efficient *PANX1* 5′ leader-containing transcript variant

**Fig. 3:**
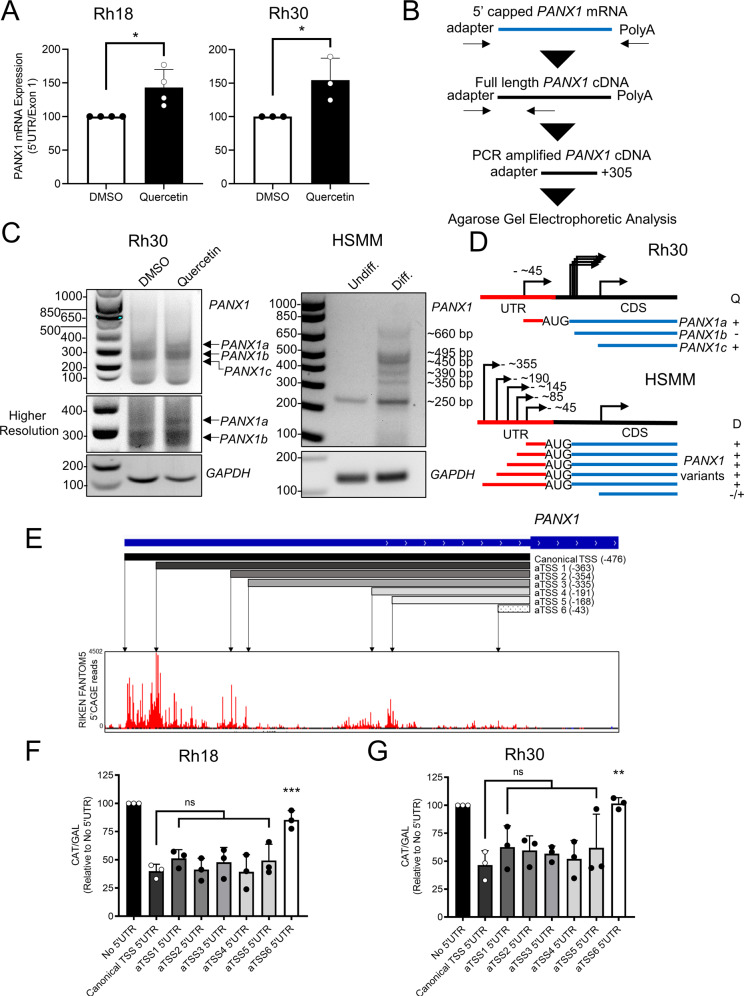
Quercetin induces the transcription of a skeletal muscle-relevant translation-competent variant of 5′ UTR-containing *PANX1* mRNA in RMS cells. **A** RT-qPCR analysis (*n* = 3 or 4) of *PANX1* transcript levels using primers specific to its 5′ UTR region from DMSO- or quercetin-treated Rh18 (eRMS) and Rh30 (aRMS) cells. Results are relative to *GAPDH* transcript levels. Statistical analysis was performed using a paired Student’s *t* test. *P < 0.05 compared to DMSO. qPCR program: 95 °C for 3 min followed by 40 cycles of 95 °C for 15 s, 60 °C for 30 s. **B** Schematic representation of the RACE workflow and regions amplified by PCR. A PCR product of 305 bps was expected for an intact *PANX1* cDNA from its ATG start codon. **C** Agarose gel electrophoresis analysis (*n* = 2) of the RACE products from Rh30 cells 24 h post-quercetin treatment or its DMSO control and HSMM before and 48 h after myogenic differentiation. A higher resolution of the agarose gel image highlighting the 5′ UTR-containing *PANX1* mRNA variant (*PANX1a*) in Rh30 cells is shown below. GAPDH was used as control for both Rh30 cells and HSMM. **D** Schematic interpretation of the RACE products from (**C**). In Rh30 cells (upper panel) the 5′ UTR-containing *PANX1* cDNA (*PANX1a*) and a truncated transcript variant (*PANX1c*) induced by quercetin treatment (Q) are shown in addition to the quercetin-independent transcript variants collectively named *PANX1b*. In HSMM (lower panel), a RACE product similar in size to *PANX1a* from quercetin-treated Rh30 cells and several other products are present under differentiating conditions (**D**). **E** Aggregated CAGE reads mapped to the 5′ UTR region of *PANX1* showing the canonical as well as six alternative transcription start sites (TSS). Data were retrieved from Riken FANTOM 5 track hub on hg38 genome and reviewed in UCSC genome browser. Translation reporter assay in Rh18 (*n* = 3) (**F**) and Rh30 (*n* = 3) (**G**) cells showing the translational activity of indicated *PANX1* putative 5′ UTRs. Plasmids encoding the indicated 5′ UTRs fused to a CAT CDS were co-transfected with β-galactosidase (GAL) to control transfection efficiency. CAT expression was measured by CAT ELISA kit, then normalized to the expression of a CAT mRNA without 5′ UTR. Statistical analysis was performed using one-way ANOVA with Tukey’s post-hoc test. **P < 0.01, ***P < 0.001 compared to the canonical TSS 5′ UTR. Results are expressed as mean ± s.d. ns not significant.

The 5′ UTR is an important regulator of translation of the downstream gene [31, 35]. By analyzing the overall ratio of PANX1, we found that the presence of transcripts containing the 5′ UTR of *PANX1* significantly increased in both quercetin-treated Rh18 and Rh30 as compared to their respective vehicle controls (Fig. 3A). To investigate the quercetin-induction of 5′ leader *PANX1* transcripts, we performed 5′RACE (Rapid Amplification of cDNA Ends) in Rh30 cells using a reverse primer designed to anneal to a position 305 bp downstream of the start codon on *PANX1* cDNA (Fig. 3B). Notably, we did not detect any discernable band at the size corresponding to the annotated *PANX1* full-length 5′ UTR (approximately 780 bp). Instead, most amplicons from both DMSO and quercetin-treated Rh30 cells were detected between 260 and 300 bp (Fig. 3C). This was consistent with the lack of 5’ UTR reads in our RNA-seq data. We termed these bands collectively as *PANX1b* (Fig. 3C). Quercetin-treated Rh30 cells also showed two additional bands: one at ~350 bp (*PANX1a*) and the other at slightly higher than 200 bp (*PANX1c*) (Fig. 3C), indicating that hitherto undescribed 5′ UTR-containing *PANX1* transcript variants emerge upon quercetin treatment (Fig. 3D). Using the same 5′RACE approach, we detected a 250 bp band in both undifferentiated and differentiated HSMM, which is similar in size to *PANX1c* (Fig. 3C). But to our surprise, we also detected several other distinct bands exclusively in differentiated HSMM (Fig. 3C), including one band at 350 bp which is identical in size to *PANX1a* (Fig. 3C, D). Using the public TSS database defined by CAGE/nanoCAGE (FANTOM Consortium and the RIKEN PMI and CLST [DGT] 2014), we found 6 other putative alternative transcription start sites (aTSS) from the annotated TSS for the *PANX1* gene locus that generated sequentially shorter 5′ UTRs, numbered as aTSS1-6 (Fig. 3E). Next, we assessed these aTSSs in a chloramphenicol acetyl transferase (CAT) translation reporter system [36]. Unexpectedly, the full-length *PANX1* 5′ UTR from the canonical TSS showed a strong repression on CAT reporter expression as compared to a 5′ UTR-less construct. The shorter alternative 5′ UTRs showed no improvement in CAT reporter activity until reaching the aTSS6 with the 43 bp-5′ UTR upstream of the start codon which dramatically increased CAT reporter activities in both Rh18 (Fig. 3F) and Rh30 (Fig. 3G) cells. Taken together, our data suggest that quercetin enhances *PANX1* translation by inducing the expression of an alternative *PANX1* transcript with a 43 bp long 5′ UTR that may also contribute to the high PANX1 levels found in differentiated HSMM.

### CREB and ETV4 binding sites within the *PANX1* promoter regulate its 5′ UTR expression

**Fig. 4:**
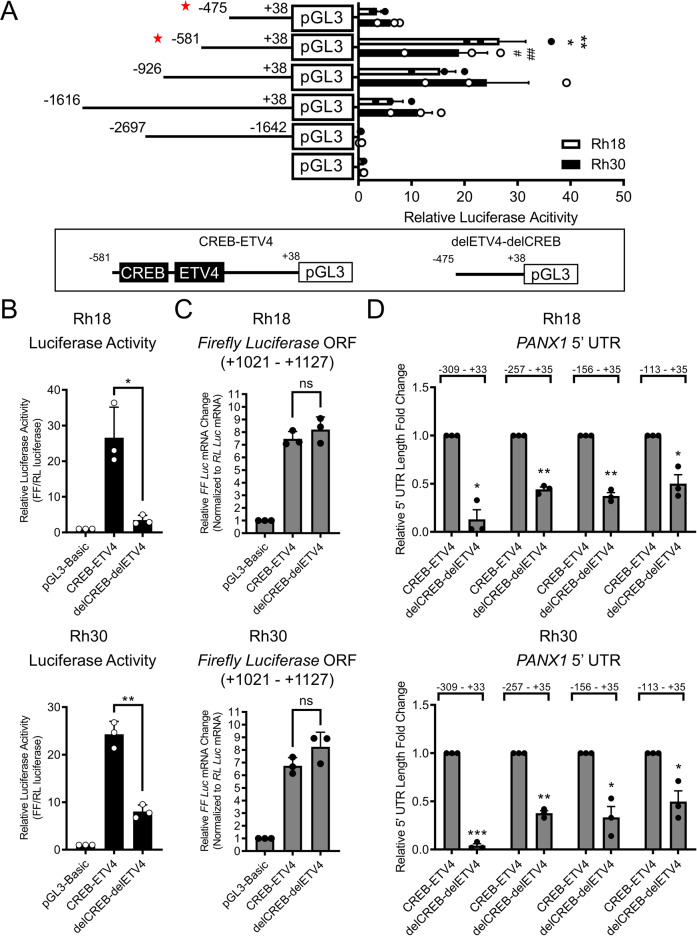
A region of the *PANX1* promoter containing CREB and ETV4 consensus sites regulates transcription of its 5′ UTR. *PANX1* promoter fragment from −2697 to −1642 as well as promoter fragments from −1616, −926, −581, and −475 to +38 relative to the ATG start codon were PCR amplified from primary HSMM cells and subcloned into the pGL3-basic plasmid. The pGL3 plasmids with *PANX1* promoter fragments were then co-transfected with pRL-TK into Rh18 (eRMS) and Rh30 (aRMS) for 48 h prior to downstream assays. **A** Luciferase activities from pGL3 carrying various portions of the *PANX1* promoter in Rh18 (*n* = 3) and Rh30 (*n* = 3) cells relative to pRL-TK. Red stars highlight the two *PANX1* promoter clones shown in the schematic diagrams below which were selected for further analyses. Statistical analysis was performed using one-way ANOVA with Tukey’s post-hoc test. *, ^#^*P* < 0.05 compared to −1616 to + 38 and **, ^##^*P* < 0.01 compared to −475 to + 38. The pGL3-Basic or pGL3 carrying *PANX1* promoter fragments from −581 to + 38 (CREB-ETV4) or −475 to + 38 (delETV4-delCREB) were co-transfected with pRL-TK into Rh18 and Rh30 cells for 48 h and subjected to dual-luciferase reporter assay (*n* = 3) (**B**) and RT-qPCR analyses (*n* = 3) for (**C**) *Firefly Luciferase* (*FF*) mRNA and (**D**) *PANX1* 5′UTR levels. Statistical analysis was performed using one-way ANOVA with Tukey’s post-hoc test for (**B**) and (**C**), while unpaired Student’s *t* tests were used in (**D**). *P < 0.05 and **P < 0.01 in (**B**). ns indicates not significant in (**C**). Each indicated amplified region was normalized separately to CREB-ETV4 in (**D**) where *P < 0.05, **P < 0.01 and ***P < 0.001 compared to their respective ETV4-CREB controls. Results are expressed as mean ± s.d.

Our results from dual-luciferase reporter assays showed no change in luciferase activities from the *PANX1* promoter section −2697 to −1642 (Fig. 4A). However, sequential removal of nucleotides at the 5′ end of *PANX1* promoter section −1616 to +38 caused a gradual increase in luciferase activity that peaked at position -581, which was almost completely abolished by a further removal of 106 bp to position −475 in both Rh18 and Rh30 cells (Fig. 4A). Interestingly, embedded in this 106 bp sequence were putative binding sites for CREB and ETV4 which were previously shown to regulate *Panx1* transcription in rat epididymal tissue [26]. Therefore, constructs containing the *PANX1* promoter sequence from positions -581 (CREB-ETV4) and −475 (delCREB-delETV4) were chosen for further analyses (Fig. 4A). delCREB-delETV4 caused a dramatic reduction of luciferase activities in Rh18 and Rh30 cells as compared to those containing CREB-ETV4 (Fig. 4B), while both *PANX1* promoter constructs produced comparable levels of *Firefly Luciferase* transcripts in Rh18 and Rh30 cells (Fig. 4C), suggesting a reduction at the translational level. We further compared its expression levels from both *PANX1* promoter constructs in Rh18 and Rh30 cells and observed a significant reduction in the *PANX1* 5′ UTR levels measured at positions −309, −257, −156, and −113 relative to the ATG in the absence of CREB and ETV4 sites as compared to their counterparts (Fig. 4D). Collectively, our data indicate that a 106 bp portion of the *PANX1* promoter containing consensus sites for the transcription factors CREB and ETV4 is important for *PANX1* 5′ UTR expression, which allows translation of the downstream coding sequence.

### PANX1 Expression in RMS Requires CREB and ETV4 Consensus Sites

**Fig. 5:**
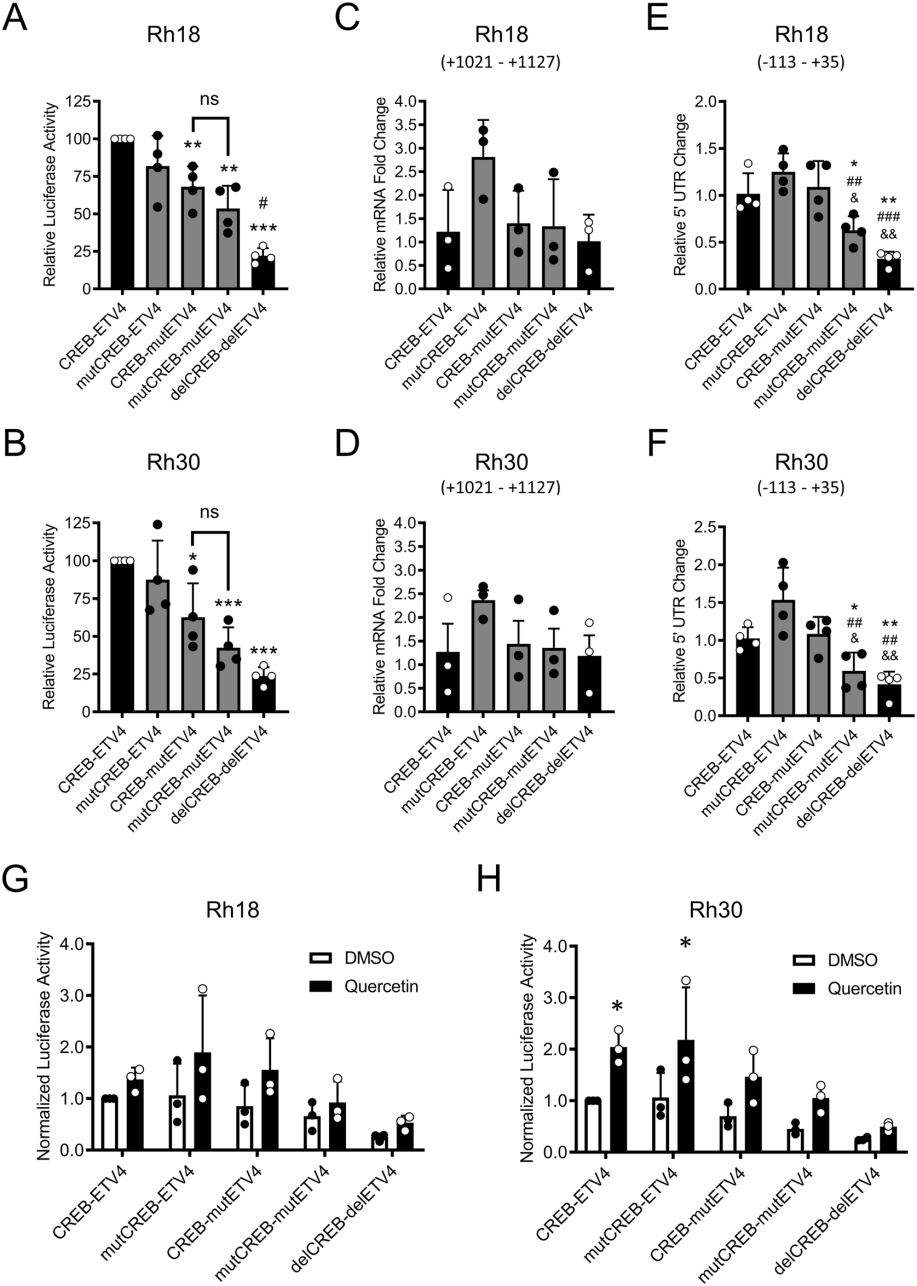
CREB and ETV4 consensus sites in the *PANX1* promoter regulate PANX1 expression in RMS cells. Rh18 (eRMS) and Rh30 (aRMS) cells were co-transfected with pRL-TK and pGL3 vectors containing one of the following constructs: the wildtype *PANX1* promoter (CREB-ETV4) or the *PANX1* promoter with point mutations in CREB (mutCREB-ETV4), ETV4 (CREB-mutETV4), or both sites (mutCREB-mutETV4). The pGL3 vector containing *PANX1* promoter with dual site deletion (delCREB-delETV4) was also included as a negative control. After 48 h, cells were analyzed by dual-luciferase reporter assays (*n* = 4) (**A**, **B**) and then with RT-qPCR (*n* = 3) for *FF* (amplified at +1021 to +1127 from *FF* ATG start codon) (**C**, **D**) or *PANX1* 5′ UTR (amplified at −113 to +35 from *PANX1* ATG start codon) (**E**, **F**) (*n* = 4). Dual-luciferase reporter assays (*n* = 3) were also performed in cells 48 h post-co-transfection following 24-h DMSO or quercetin treatment prior to analysis (**G**, **H**). One-way ANOVA with Tukey’s post-hoc test was performed in (**A**–**F**). In (**A**) and (**B**), *P < 0.05, **P < 0.01 and ***P < 0.001 compared to CREB-ETV4; ^#^*P* < 0.05 compared to mutCREB-mutEVT4; ns = not significant. In (**E**) and (**F**), *P < 0.05 and **P < 0.01 compared to CREB-ETV4; ^##^*P* < 0.01 and ^###^*P* < 0.001 compared to mutCREB-ETV4; ^&^*P* < 0.05 and ^&&^*P* < 0.01 compared to CREB-mutETV4. Multiple Student’s *t* tests with Holm–Sidak correction for multiple comparisons were performed in (**G**, **H**). In (**H**), *P < 0.05 compared to DMSO. pRL-TK was used as an internal normalizer in all results shown. Results are expressed as mean ± s.d. (*n* = 3 or 4).

Next, site-directed mutagenesis was performed to disrupt the putative consensus nucleotide sequences in the CREB binding site alone (mutCREB-ETV4) or the ETV4 binding site alone (CREB-mutETV4) or both CREB and ETV4 binding sites (mutCREB-mutETV4) from the CREB-ETV4 *PANX1* promoter construct in pGL3 vector. Interestingly, while the CREB site mutation alone did not change luciferase activities, the ETV4 site mutation alone (CREB-mutETV4) and the CREB-ETV4 dual site mutation (mutCREB-mutETV4) both induced similar yet significant reductions in luciferase activities as compared to the positive controls in Rh18 (Fig. 5A) and Rh30 (Fig. 5B). Similar to previous findings, none of the *PANX1* promoter site-mutation or deletion constructs significantly changed the levels of *Firefly Luciferase* reporter gene transcript in both Rh18 (Fig. 5C) and Rh30 (Fig. 5D) cells. Moreover, dual CREB-ETV4 site mutation also significantly reduced the 5′ portion of the *Firefly Luciferase* reporter gene transcript that contained the *PANX1* 5′ UTR in Rh18 (Fig. 5E) and Rh30 (Fig. 5F) cells. Our further dual-luciferase reporter assays showed a trend towards a moderate increase in luciferase activities in all *PANX1* promoter constructs used after quercetin treatment in Rh18 cells (Fig. 5G). However, we observed a significant increase in luciferase activities following quercetin treatment with CREB-ETV4 and mutCREB-ETV4 constructs in Rh30 cells (Fig. 5H). This increase was no longer statistically significant when the ETV4 site was mutated or CREB and ETV4 sites were both deleted (Fig. 5H). Luciferase activity was measured 48 h after transfection with quercetin treatment in the last 24 h. No changes in both *Firefly Luciferase and PANX1* 5′UTR transcript expression were detected between 24 and 48 h (data not shown) suggesting that these transcripts had reached a steady state 24 h after transfection and indicating that the induction of luciferase activity observed was due to quercetin treatment alone. Altogether, these results further suggest the involvement of transcription factors CREB and ETV4 in regulating the transcription of *PANX1* from its 5′UTR which controls its translation.

### ETV4 binding to its consensus site in the *PANX1* promoter is enhanced in quercetin-treated Rh30 cells and during myogenic differentiation

**Fig. 6:**
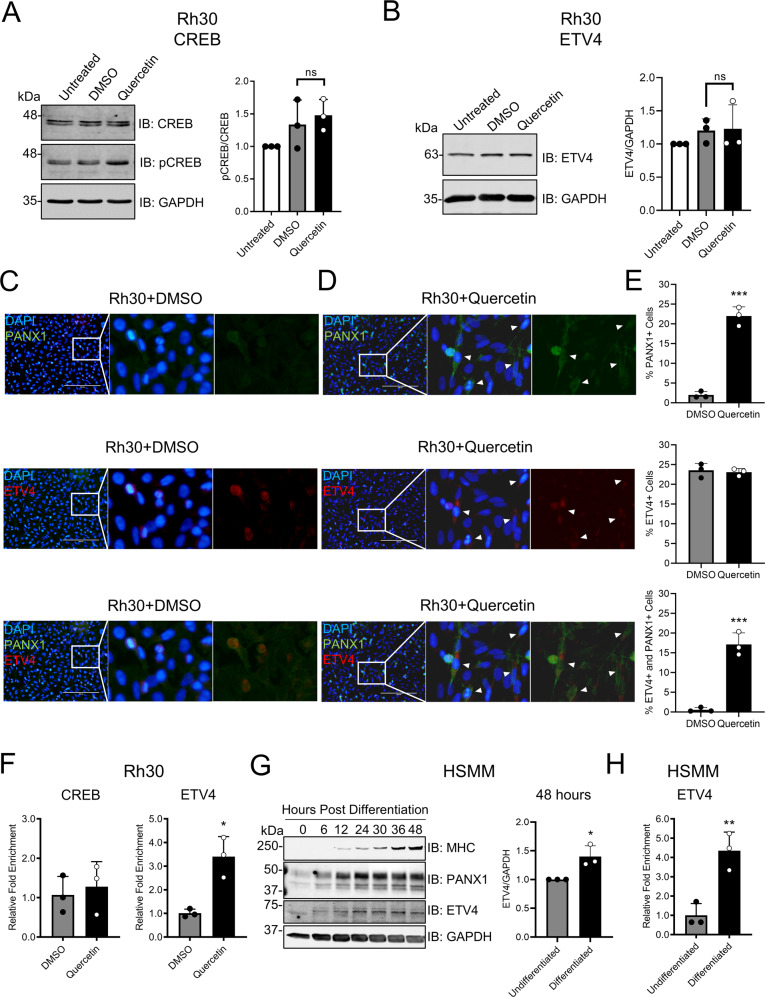
Quercetin enhances the binding of ETV4 to its consensus site in the *PANX1* promoter. Rh30 (aRMS) cells were treated with quercetin or DMSO for 24 h and then subjected to various analyses. Representative Western blots and their respective quantification (*n* = 3) for CREB and pCREB (**A**) and ETV4 (**B**). GAPDH was used as a loading control. Results were normalized to untreated Rh30 cells. One-way ANOVA with Tukey’s post-hoc test was performed in (**A**, **B**). ns = not significant. **C**, **D** Representative immunofluorescence (IF) images showing DAPI (blue), PANX1 (green) and ETV4 (red). Images were taken with a 20X objective. Arrowheads indicate PANX1 and ETV4 dual-positive Rh30 cells. Bar = 200 µm. **E** Quantification of PANX1 and ETV4 expressing Rh30 cells from (**C**, **D**). Results are expressed as mean percent (%) cells from five random fields (*n* = 3). ***P < 0.001 compared to DMSO. **F** Chromatin Immunoprecipitation (ChIP) of CREB and ETV4 in Rh30 cells (*n* = 3) showing enrichment of transcriptional factor binding to their respective consensus site in the *PANX1* promoter. *P < 0.05 compared to DMSO. **G** Representative Western blot showing the change of MHC, PANX1, and ETV4 levels during the first 48 h of HSMM differentiation and the quantification (*n* = 3) of ETV4 levels at 48 h post-myogenic differentiation. *P < 0.05 compared to Undifferentiated. **H** ChIP assay results (*n* = 3) of undifferentiated and differentiated HSMM. HSMM cells were induced to differentiate by serum-withdrawal for 6 days prior to analysis. **P < 0.01 compared to Undifferentiated. Results are expressed as mean ± s.d. Paired Student’s *t* test was performed in **(E**–**H)**.

Both total CREB and phosphorylated CREB (ser133) were detected in Rh30 cells (Fig. 6A), but phosphorylated CREB levels following quercetin treatment were similar to the control (Fig. 6A). Although quercetin treatment did not increase ETV4 levels in Rh30 cells (Fig. 6B), only quercetin-treated Rh30 cells showed clear co-labeling of PANX1 (green) and ETV4 (red) (Fig. 6C, D). While quercetin treatment did not change the proportion of ETV4-positive cells compared to that of the DMSO controls, it resulted in a dramatic, yet expected, increase in the number of PANX1-positive cells as well as a significant increase in the proportion of cells that were positive for both PANX1 and ETV4 (Fig. 6E). More importantly, although subsequent chromatin immunoprecipitation (ChIP) assays showed no change in CREB binding to its consensus site on the *PANX1* promoter in quercetin-treated Rh30 cells as compared to their DMSO controls (Fig. 6F, left panel), ETV4 binding was significantly enriched by quercetin (Fig. 6F, right panel). As PANX1 levels were highly upregulated during HSMM differentiation [6], we suspected that ETV4 binding to the *PANX1* promoter would also be enriched during this process. Interestingly, ETV4 expression started to increase concomitantly with PANX1 levels in HSMM at 6 h, and became significantly elevated at 48 h, following induction of differentiation (Fig. 6G). Moreover, the ChIP assay showed a clear enrichment of ETV4 binding to the *PANX1* promoter in differentiated HSMM as compared to undifferentiated cells (Fig. 6H). Collectively, these data suggest that the mechanism by which quercetin upregulates *PANX1* translation in RMS cells involves the binding of ETV4, independent of its expression, to its promoter region and the transcription of a translation-efficient 5′leader-containing *PANX1* mRNA.

### Quercetin induces partial myogenic differentiation of RMS cells in a PANX1-dependent manner, and prevents formation and induces regression of established 3D in vitro tumors

**Fig. 7:**
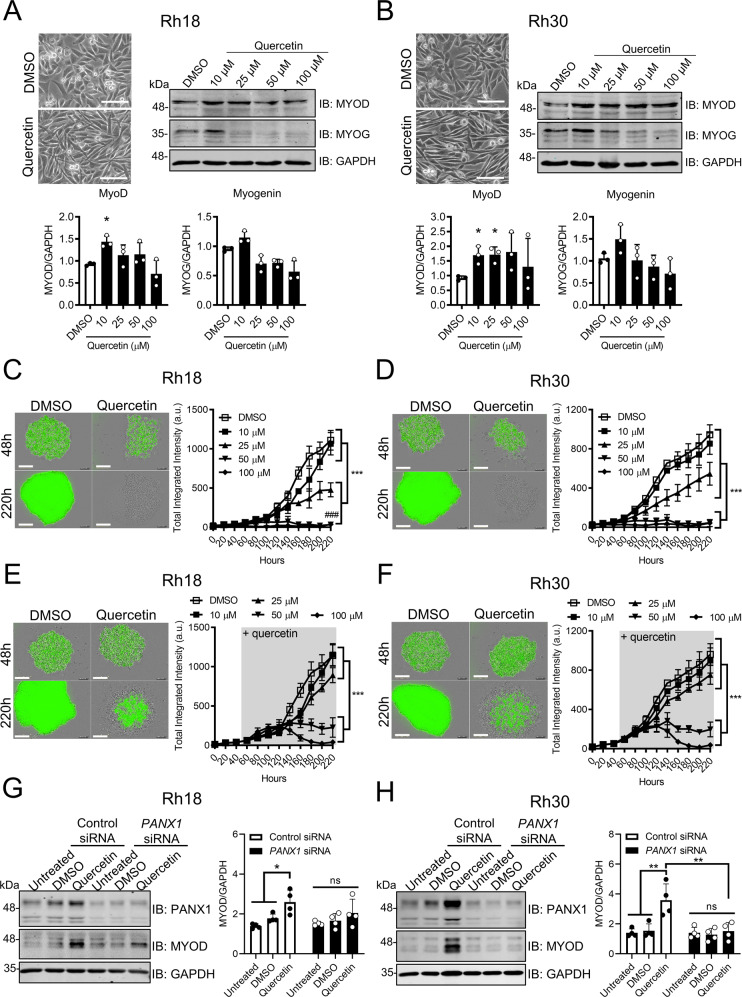
Quercetin induces partial differentiation of RMS cells in a PANX1-dependent manner, and both inhibits formation of and induces regression of their established 3D in vitro tumors. Representative images, Western blots, and their quantifications for myogenic markers, MyoD and MYOG, from Rh18 (eRMS) (*n* = 3) (**A**) and Rh30 (aRMS) (*n* = 3) (**B**) cells 24 h post-quercetin treatment. DMSO was used as vehicle control. Microscopic images show morphological changes of cells treated with 10 μM quercetin for 24 h. Bar = 100 µm. GAPDH was used as a loading control. *P < 0.05 compared to DMSO. Rh18 and Rh30 cells stably expressing GFP were subjected to 3D spheroid formation and regression assays. The GFP fluorescence, a surrogate measurement of spheroid size, was monitored over 220 h. In 3D spheroid formation and growth assay, Rh18 and Rh30 cells were pre-treated with quercetin or DMSO for 24 h. Representative images of Rh18 and Rh30 cells treated with 50 µM quercetin at 48 and 220 h are shown. The changes in total integrated intensities for Rh18 (*n* = 3) (**C**) and Rh30 (*n* = 3) (**D**) spheroids treated with the indicated dosages of quercetin are summarized to the right. In 3D spheroid regression assays, Rh18 and Rh30 cells were treated with quercetin 48 h post-spheroid formation. Representative images of Rh18 (**E**) and Rh30 (**F**) cells treated with 50 µM of quercetin at 48 h (the time of treatment initiation) and 220 h are shown. Changes in total integrated intensities of Rh18 (*n* = 3) (**E**) and Rh30 (*n* = 3) (**F**) spheroids treated with the indicated dosages of quercetin are summarized to the right. ***P < 0.001 between indicated groups. ^###^*P* < 0.001 compared to 25 µM. Bar = 300 µm. Rh18 and Rh30 cells were transiently transfected with control or *PANX1*-targeting siRNA for 72 h and treated with 10 μM quercetin for an additional 24 h. Representative Western blots and their corresponding quantifications from Rh18 (*n* = 4) (**G**) and Rh30 (*n* = 4) (**H**) show partial and complete rescue of MyoD levels, respectively. *P < 0.05 compared to Control siRNA. Results are expressed as mean + or ± s.d. One-way and two-way ANOVA with Tukey’s post-hoc tests were performed in (**A**–**F**) and (**G**–**H**), respectively. In (**C**–**F**), only the Total Integrated Intensities at the endpoints were compared.

Since quercetin has been shown to suppress various malignancies [32, 37–39], we next wanted to determine whether quercetin could also induce tumor-suppression in RMS cells. Following 24-hour exposure to quercetin, both Rh18 (Fig. 7A) and Rh30 (Fig. 7B) cells became elongated as compared to their respective controls, which is indicative of myogenic differentiation [8]. Consistent with this, subsequent Western blotting further showed a significant upregulation of MyoD levels and a trend towards an increase in MYOG after treatment with quercetin at 10 µM in both Rh18 and Rh30 cells (Fig. 7A, B). Next, we assessed the effect of quercetin in Rh18 and Rh30 3D spheroid formation and regression [12]. Quercetin treatment induced a dose-dependent inhibitory effect on Rh18 (Fig. 7C) and Rh30 (Fig. 7D) spheroid formation and growth, completely abolishing their formation at the 50–100 µM range. Similarly, when Rh18 (Fig. 7E) and Rh30 (Fig. 7F) cells were exposed to quercetin after having been allowed to develop into established spheroids, a dramatic dose-dependent regression was observed. We then investigated the role of PANX1 in quercetin-induced RMS tumor suppression by genetically targeting *PANX1* using siRNA. Following PANX1 knockdown in Rh18 cells, quercetin induced a slight, no longer statistically significant, increase in MyoD levels (Fig. 7G). Notably, quercetin treatment completely failed to induce MyoD expression in Rh30 cells when PANX1 was knocked down, resulting in significantly lower MyoD levels compared to those in its respective control (Fig. 7H). Together, these data demonstrate the tumor-suppressive effects of quercetin in RMS and support its potential use as a therapeutic agent for this malignancy.

## Discussion

**Fig. 8:**
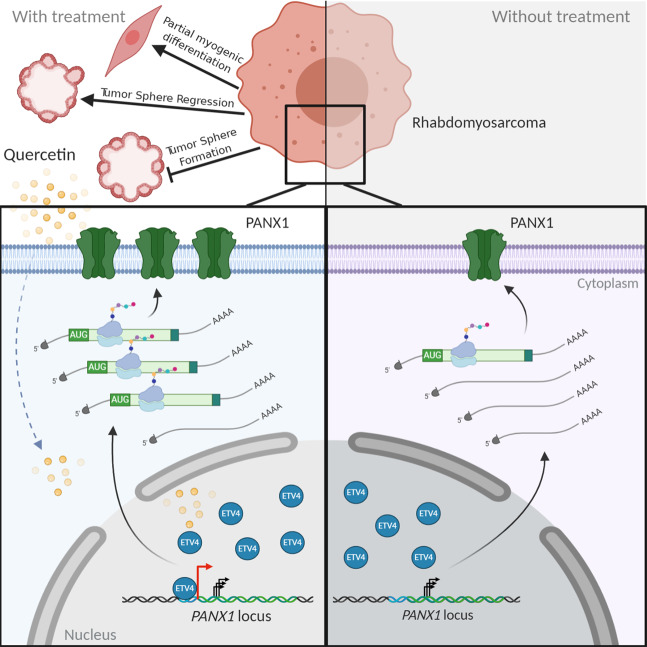
Quercetin-induced transcriptional and translational control of *PANX1* in RMS. Under normal culture conditions, the majority of basal *PANX1* transcription starts after the ATG start codon generating truncated mRNA of varying lengths that are not readily translated into PANX1 protein. Quercetin treatment enhances the binding of ETV4 to its consensus site in *PANX1* promoter, which allows the transcription of a variant of the *PANX1* transcript containing a short (~43 bp) fragment of its putative 5′ UTR. This 5′ UTR-containing variant of the full-length *PANX1* transcript can be readily bound by ribosomes resulting in an enhancement in its translation. This results in an increase in PANX1 levels and alleviation of RMS malignant properties, which suggests that repurposing of quercetin for RMS may constitute a potential new therapeutic strategy for this neoplasm. Moreover, the switch in *PANX1* transcription from producing various translationally incompetent mRNA to a more translatable 5′ UTR-containing variant of mRNA may have implications in the rapid modulation of PANX1 levels observed during tissue development and homeostasis, and by contrast, the dysregulation of which may lead to pathological conditions.

We present here a correlation between the levels of 5′ UTR-containing *PANX1* transcripts and PANX1 protein in skeletal muscle and RMS. We show that quercetin increases PANX1 levels in RMS cells by enriching the binding of the transcription factor ETV4 to the *PANX1* promoter, which induces the expression of an alternative *PANX1* mRNA transcript variant containing a translationally competent 5′ leader. Moreover, we also demonstrate the tumor-suppressive effects of quercetin in RMS and the possibility for future clinical translation (Fig. 8).

Our initial RNA-seq and RT-qPCR data revealed a unique transcriptional profile of *PANX1* in RMS where most of the transcription starts sporadically around the ATG start codon without a defined TSS. The *PANX1*/*Panx1* promoters have been shown to reside in CpG islands and lack core promoter elements [26]. Such promoters generally have dispersed transcription initiation [40, 41]. Our data also suggest a considerable basal level of *PANX1* transcription in RMS cells. However, due to the lack of the *PANX1* 5′ UTR, or in most cases the ATG start codon, they are deficient in the necessary message for translation into functional full-length PANX1 protein. Indeed, PANX1 levels in RMS are significantly lower than those of differentiated HSMM. Skeletal muscle differentiation is an intricately regulated process which involves precise temporal regulation of genes including *PANX1* [6, 8]. Thus, it is not surprising to see such aberrant transcription of *PANX1* in RMS as their myogenic differentiation program is largely impaired [42]. Our observation adds to a growing body of literature that has identified the dynamic regulation of transcript variants, not just transcript abundance, to play an important role in eukaryotic gene expression via altering the translational output of each transcript [43, 44].

RNA-seq data from Hadwen et al. (2018) showed that quercetin induced *Panx1* mRNA levels in murine primary cerebral cultures. As opposed to an induction of *PANX1* mRNA levels, quercetin induced PANX1 protein expression at the translational level in RMS. While total *PANX1* mRNA levels stayed unchanged, we detected an increase of transcripts containing a 5′leader region, suggesting possible alternative transcription of *PANX1*. Our results from 5′RACE of *PANX1* in Rh30 cells are also in accordance with our alignment of RNA-seq reads which reveal a broad band between ~260 to 300 bp indicative of *PANX1* transcripts with 5′ truncation. However, we also identified two additional distinct bands following quercetin treatment that are ~350 bp (*PANX1a*) and ~230 bp (*PANX1c*) long. While *PANX1b* and *PANX1c* are truncated beyond the first ATG start codon, *PANX1a* represents a potentially translatable full-length mRNA. Interestingly, a ~350 bp band similar to *PANX1a* was exclusively detected in differentiated HSMM that are known to express abundant levels of PANX1 [6], which not only may potentially be the same *PANX1a* variant in Rh30 cells but also suggests its translational competence. Notably, our survey of various *PANX1* 5′UTR lengths (between position −476 and −43 relative to the ATG start codon), which represent multiple potential alternative TSSs based on aggregated CAGE data, shows that the 5′ UTR from *PANX1a* (43 bp) is the most translationally active. Furthermore, we also showed, for the first time, that multiple other alternative *PANX1* transcripts exist in HSMM during myogenic differentiation, which correspond to potential aTSSs at −85, −145, −190, and −355 bp upstream to the ATG start codon. Remarkably, these aTSS are a perfect match, or within a few bases, relative to those predicted *PANX1* aTSSs from aggregated CAGE results. Alternative transcription of *Panx1* has been reported in rat epididymis which leads to three different *Panx1* mRNA transcript variants with 393 bp-, 429 bp- and 443 bp-long 5′UTR, respectively [26]. Although the translational activities of these *Panx1* 5′ UTRs were not studied in rat epididymis, their protein product Panx1 has been detected in the same tissue [45] suggesting that they are translationally active. These data indicate potential tissue-dependent translational activity of the *PANX1* 5′ UTR resulting in dynamic regulation of PANX1 expression [6, 19–21].

The *PANX1* promoter is evolutionarily conserved between human and rodents especially in the region containing consensus sites for CREB and ETV4 [26]. Interestingly, we find that quercetin upregulates PANX1 in RMS through a mechanism of alternative transcription of *PANX1* involving ETV4. ETV4 has been detected in skeletal muscle and shown to be critically involved in myogenic differentiation [46]. The transactivity of ETV4 is enhanced by MEF2 (myocyte enhancer factor 2C) and, more importantly, over-expression of ETV4 accelerates myogenic differentiation and blocking ETV4 function inhibits this process [46, 47]. Our data add to the existing evidence by further showing the simultaneous increase of PANX1 and ETV4 levels and enrichment of ETV4 binding to the *PANX1* promoter during HSMM differentiation, thus linking the functional role of ETV4 to one of its downstream effector genes, *PANX1*, in myogenic differentiation [6] and skeletal muscle regeneration [7, 46, 48]. Similar to PANX1, ETV4 expression in RMS is generally low [49]. This correlation between ETV4 and PANX1 levels also remains in breast cancer [50, 51], prostate cancer [52, 53], and melanoma [24, 54], which further broadens the contextual implications of ETV4 for PANX1 expression. Our data also suggests the involvement of other transcription factors as abolishing the ETV4 binding site does not completely prevent the increase in reporter activity by quercetin. One potential candidate is interferon response factor 2 (IRF-2), an important regulator of skeletal myogenesis [55], as removal of its consensus site in the *PANX1* promoter significantly reduced reporter gene transcript levels and activities (data not shown). Thus, future efforts will be directed to deciphering the role of IRF-2 or other potential TFs on *PANX1* promoter regulation.

Moreover, our findings further expand the current functional implications of quercetin by suggesting that it may constitute a potential new therapeutic agent for diseases such as RMS [12], glioma [22], and hearing loss [56] where upregulation of PANX1 has been shown to be potentially beneficial. Notably, quercetin has various protective roles in skeletal muscle [57–59]. It has been recently shown to upregulate *MEF2C* [60], an important myogenic factor in skeletal muscle differentiation [61] that is dysregulated in RMS [62, 63]. An earlier study also reported a dose-dependent inhibition of proliferation and morphological changes in RD cells, an eRMS cell line, after 48 h in the presence of 30–130 µM of quercetin [64]. In keeping with our previous findings [12], our data suggest that PANX1 plays a part in quercetin-induced partial differentiation of RMS cells. Moreover, quercetin treatment resulted in a dose-dependent growth inhibition and regression of Rh18 and Rh30 3D tumor spheroids at concentrations well tolerated by normal human cells [65]. Together, our findings warrant future studies to further assess the tumor-suppressive effects of quercetin in RMS with in vivo mouse models and its potential clinical translation for RMS treatment.

## Supplementary information

Supplemental Material and Methods

Supplementary Information (Table S1 - List of Primers)

## References

[1] Ruan Z, Orozco IJ, Du J, Lü W. Structures of human pannexin 1 reveal ion pathways and mechanism of gating. Nature. 2020; 10.1038/s41586-020-2357-y. 10.1038/s41586-020-2357-y PMC7814660 32494015

[2] Deng Z, He Z, Maksaev G, Bitter RM, Rau M, Fitzpatrick JAJ (2020). Cryo-EM structures of the ATP release channel pannexin 1. Nat Struct Mol Biol.

[3] Jin Q, Zhang B, Zheng X, Li N, Xu L, Xie Y (2020). Cryo-EM structures of human pannexin 1 channel. Cell Res.

[4] Michalski K, Syrjanen JL, Henze E, Kumpf J, Furukawa H, Kawate T (2020). The Cryo-EM structure of a pannexin 1 reveals unique motifs for ion selection and inhibition. Elife.

[5] Bao L, Locovei S, Dahl G (2004). Pannexin membrane channels are mechanosensitive conduits for ATP. FEBS Lett.

[6] Langlois S, Xiang X, Young K, Cowan BJ, Penuela S, Cowan KN (2014). Pannexin 1 and pannexin 3 channels regulate skeletal muscle myoblast proliferation and differentiation. J Biol Chem.

[7] Pham TL, St-Pierre ME, Ravel-Chapuis A, Parks TEC, Langlois S, Penuela S (2018). Expression of Pannexin 1 and Pannexin 3 during skeletal muscle development, regeneration, and Duchenne muscular dystrophy. J Cell Physiol.

[8] Langlois S, Cowan KN (2017). Regulation of skeletal muscle myoblast differentiation and proliferation by pannexins. Adv Exp Med Biol.

[9] Lav R, Heera R, Cherian LM (2015). Decoding the ‘embryonic’ nature of embryonal rhabdomyosarcoma. J Dev Orig Health Dis.

[10] Charytonowicz E, Cordon-Cardo C, Matushansky I, Ziman M (2009). Alveolar rhabdomyosarcoma: Is the cell of origin a mesenchymal stem cell?. Cancer Lett.

[11] Monti E, Fanzani A (2015). Uncovering metabolism in rhabdomyosarcoma. Cell Cycle.

[12] Xiang X, Langlois S, St-Pierre ME, Barré JF, Grynspan D, Purgina B, et al. Pannexin 1 inhibits rhabdomyosarcoma progression through a mechanism independent of its canonical channel function. Oncogenesis. 2018;7. 10.1038/s41389-018-0100-4. 10.1038/s41389-018-0100-4 PMC6246549 30459312

[13] Xiang X, Langlois S, St-Pierre ME, Blinder A, Charron P, Graber TE, et al. Identification of pannexin 1-regulated genes, interactome, and pathways in rhabdomyosarcoma and its tumor inhibitory interaction with AHNAK. Oncogene. 2021;1. 10.1038/s41388-020-01623-2. 10.1038/s41388-020-01623-2 PMC7946643 33564071

[14] Amer KM, Thomson JE, Congiusta D, Dobitsch A, Chaudhry A, Li M (2019). Epidemiology, incidence, and survival of rhabdomyosarcoma subtypes: SEER and ICES database analysis. J Orthop Res.

[15] Punyko JA, Mertens AC, Baker KS, Ness KK, Robison LL, Gurney JG (2005). Long-term survival probabilities for childhood rhabdomyosarcoma. A population-based evaluation. Cancer.

[16] Oberlin O, Rey A, Lyden E, Bisogno G, Stevens MCGG, Meyer WH (2008). Prognostic factors in metastatic rhabdomyosarcomas: results of a pooled analysis from United States and European cooperative groups. J Clin Oncol.

[17] Dobson CC, Langlois S, Grynspan D, Cowan KN (2016). Engaging cell death pathways for the treatment of rhabdomyosarcoma. Crit Rev Oncog.

[18] Hettmer S, Li Z, Billin AN, Barr FG, Cornelison DDW, Ehrlich AR (2014). Rhabdomyosarcoma: current challenges and their implications for developing therapies. Cold Spring Harb Perspect Med.

[19] Ray A, Zoidl G, Weickert S, Wahle P, Dermietzel R (2005). Site-specific and developmental expression of pannexin1 in the mouse nervous system. Eur J Neurosci.

[20] Vogt A, Hormuzdi SG, Monyer H (2005). Pannexin1 and Pannexin2 expression in the developing and mature rat brain. Mol Brain Res.

[21] Lee V, Barr K, Kelly J, Johnston D, Brown C, Robb K (2018). Pannexin 1 regulates adipose stromal cell differentiation and fat accumulation. Sci Rep.

[22] Lai CPK, Bechberger JF, Thompson RJ, MacVicar BA, Bruzzone R, Naus CC (2007). Tumor-suppressive effects of pannexin 1 in C6 glioma cells. Cancer Res.

[23] Penuela S, Gyenis L, Ablack A, Churko JM, Berger AC, Litchfield DW (2012). Loss of pannexin 1 attenuates melanoma progression by reversion to a melanocytic phenotype. J Biol Chem.

[24] Freeman TJ, Sayedyahossein S, Johnston D, Sanchez-Pupo RE, O’Donnell B, Huang K (2019). Inhibition of pannexin 1 reduces the tumorigenic properties of human melanoma cells. Cancers (Basel).

[25] Boyce AKJ, Epp AL, Nagarajan A, Swayne LA (2018). Transcriptional and post-translational regulation of pannexins. Biochim Biophys Acta—Biomembr.

[26] Dufresne J, Cyr DG (2014). Regulation of the pannexin-1 promoter in the rat epididymis. Biol Reprod.

[27] Tang SM, Deng XT, Zhou J, Li QP, Ge XX, Miao L (2020). Pharmacological basis and new insights of quercetin action in respect to its anti-cancer effects. Biomed Pharmacother.

[28] Hadwen J, Schock S, Mears A, Yang R, Charron P, Zhang L (2018). Transcriptomic RNAseq drug screen in cerebrocortical cultures: toward novel neurogenetic disease therapies. Hum Mol Genet.

[29] Hinson ARP, Jones R, Crose LES, Belyea BC, Barr FG, Linardic CM (2013). Human rhabdomyosarcoma cell lines for rhabdomyosarcoma research: utility and pitfalls. Front Oncol.

[30] Cowan KN, Langlois S, Penuela S, Cowan BJ, Laird DW (2012). Pannexin1 and Pannexin3 exhibit distinct localization patterns in human skin appendages and are regulated during keratinocyte differentiation and carcinogenesis. Cell Commun Adhes.

[31] Araujo PR, Yoon K, Ko D, Smith AD, Qiao M, Suresh U (2012). Before it gets started: regulating translation at the 5’ UTR. Comp Funct Genomics.

[32] Rauf A, Imran M, Khan IA, ur-Rehman M, Gilani SA, Mehmood Z (2018). Anticancer potential of quercetin: a comprehensive review. Phyther Res.

[33] Penuela S, Lohman AW, Lai W, Gyenis L, Litchfield DW, Isakson BE (2014). Diverse post-translational modifications of the pannexin family of channel-forming proteins. Channels (Austin).

[34] Gandin V, Sikström K, Alain T, Morita M, McLaughlan S, Larsson O (2014). Polysome fractionation and analysis of mammalian translatomes on a genome-wide scale. J Vis Exp.

[35] Hinnebusch AG, Ivanov IP, Sonenberg N (2016). Translational control by 5’-untranslated regions of eukaryotic mRNAs. Science.

[36] Graber TE, Baird SD, Kao PN, Mathews MB, Holcik M (2010). NF45 functions as an IRES transacting factor that is required for translation of cIAP1 during the unfolded protein response. Cell Death Differ.

[37] Cossarizza A, Gibellini L, Pinti M, Nasi M, Montagna JP, De Biasi S, et al. Quercetin and cancer chemoprevention. Evidence-based Complement Altern Med. 2011;2011. 10.1093/ecam/neq053. 10.1093/ecam/neq053 PMC3136711 21792362

[38] Reyes-Farias M, Carrasco-Pozo C (2019). The anti-cancer effect of quercetin: molecular implications in cancer metabolism. Int J Mol Sci.

[39] Shafabakhsh R, Asemi Z (2019). Quercetin: a natural compound for ovarian cancer treatment. J Ovarian Res.

[40] Zhang L, Yu H, Wang P, Ding Q, Wang Z (2013). Screening of transcription factors with transcriptional initiation activity. Gene.

[41] Butler JEF, Kadonaga JT (2002). The RNA polymerase II core promoter: a key component in the regulation of gene expression. Genes Dev.

[42] Keller C, Guttridge DC (2013). Mechanisms of impaired differentiation in rhabdomyosarcoma. FEBS J.

[43] Floor SN, Doudna JA (2016). Tunable protein synthesis by transcript isoforms in human cells. Elife.

[44] Cheng Z, Otto GM, Powers EN, Keskin A, Mertins P, Carr SA (2018). Pervasive, coordinated protein-level changes driven by transcript isoform switching during meiosis. Cell.

[45] Turmel P, Dufresne J, Hermo L, Smith CE, Penuela S, Laird DW (2011). Characterization of pannexin1 and pannexin3 and their regulation by androgens in the male reproductive tract of the adult rat. Mol Reprod Dev.

[46] Taylor JM, Dupont-Versteegden EE, Davies JD, Hassell JA, Houlé JD, Gurley CM (1997). A role for the ETS domain transcription factor PEA3 in myogenic differentiation. Mol Cell Biol.

[47] Sabourin LA, Girgis-Gabardo A, Seale P, Asakura A, Rudnicki MA (1999). Reduced differentiation potential of primary MYOD−/− myogenic cells derived from adult skeletal muscle. J Cell Biol.

[48] Suarez-Berumen K, Collins-Hooper H, Gromova A, Meech R, Sacco A, Dash PR, et al. Pannexin 1 regulates skeletal muscle regeneration by promoting bleb-based myoblast migration and fusion through a novel lipid based signaling mechanism. Front Cell Dev Biol. 2021;9. 10.3389/fcell.2021.736813. 10.3389/fcell.2021.736813 PMC8523994 34676213

[49] Le Guellec S, Velasco V, Pérot G, Watson S, Tirode F, Coindre JM (2016). ETV4 is a useful marker for the diagnosis of CIC-rearranged undifferentiated round-cell sarcomas: a study of 127 cases including mimicking lesions. Mod Pathol.

[50] Qin L, Liao L, Redmond A, Young L, Yuan Y, Chen H (2008). The AIB1 oncogene promotes breast cancer metastasis by activation of PEA3-mediated matrix metalloproteinase 2 (MMP2) and MMP9 expression. Mol Cell Biol.

[51] Wu D, Li L, Chen L (2016). A new perspective of mechanosensitive pannexin-1 channels in cancer metastasis: clues for the treatment of other stress-induced diseases. Acta Biochim Biophys Sin (Shanghai).

[52] Tomlins SA, Mehra R, Rhodes DR, Smith LR, Roulston D, Helgeson BE (2006). TMPRSS2:ETV4 gene fusions define a third molecular subtype of prostate cancer. Cancer Res.

[53] Vanden Abeele F, Bidaux G, Gordienko D, Beck B, Panchin YV, Baranova AV (2006). Functional implications of calcium permeability of the channel formed by pannexin 1. J Cell Biol.

[54] Li S, Huang X, Zhang D, Huang Q, Pei G, Wang L (2013). Requirement of PEA3 for transcriptional activation of FAK gene in tumor metastasis. PLoS ONE.

[55] Jesse T, LaChance R (1998). Interferon regulatory factor-2 is a transcriptional activator in muscle where it regulates expression of vascular cell adhesion molecule-1. J Cell Biol.

[56] Zhao H-B, Zhu Y, Liang C, Chen J (2015). Pannexin 1 deficiency can induce hearing loss. Biochem Biophys Res Commun.

[57] Le NH, Kim CS, Park T, Park JHY, Sung MK, Lee DG, et al. Quercetin protects against obesity-induced skeletal muscle inflammation and atrophy. Mediators Inflamm. 2014;2014. 10.1155/2014/834294. 10.1155/2014/834294 PMC4295595 25614714

[58] Ekinci Akdemir FN, Gülçin İ, Karagöz B, Soslu R (2016). Quercetin protects rat skeletal muscle from ischemia reperfusion injury. J Enzym Inhib Med Chem.

[59] Spaulding HR, Ballmann CG, Quindry JC, Selsby JT (2016). Long-term quercetin dietary enrichment partially protects dystrophic skeletal muscle. PLoS ONE.

[60] Atrahimovich D, Samson AO, Barsheshet Y, Vaya J, Khatib S, Reuveni E (2019). Genome-wide localization of the polyphenol quercetin in human monocytes. BMC Genomics.

[61] Potthoff MJ, Arnold MA, McAnally J, Richardson JA, Bassel-Duby R, Olson EN (2007). Regulation of skeletal muscle sarcomere integrity and postnatal muscle function by Mef2c. Mol Cell Biol.

[62] Zhang M, Zhu B, Davie J (2015). Alternative splicing of MEF2C pre-mRNA controls its activity in normal myogenesis and promotes tumorigenicity in rhabdomyosarcoma cells. J Biol Chem.

[63] Ignatius MS, Hayes MN, Lobbardi R, Chen EY, McCarthy KM, Sreenivas P (2017). The NOTCH1/SNAIL1/MEF2C pathway regulates growth and self-renewal in embryonal rhabdomyosarcoma. Cell Rep..

[64] Jagadeeswaran R, Thirunavukkarasu C, Gunasekaran P, Ramamurty N, Sakthisekaran D (2000). In vitro studies on the selective cytotoxic effect of crocetin and quercetin. Fitoterapia.

[65] Matsuo M, Sasaki N, Saga K, Kaneko T (2005). Cytotoxicity of flavonoids toward cultured normal human cells. Biol Pharm Bull.

