# Supplementary material for: Quercetin induces pannexin 1 expression via an alternative transcript with a translationally active 5′ leader in rhabdomyosarcoma
Source: Oncogenesis. 2022 Feb 22;11(1):9. doi: 10.1038/s41389-022-00384-9 (PMC8864035; doi:10.1038/s41389-022-00384-9)
Supplement: Supplementary file 1 — Supplemental Material and Methods [file 41389_2022_384_MOESM1_ESM.docx]

**SUPPLEMENTAL MATERIAL AND METHODS**

## Cell Culture, Drug Treatment and Transfection

Patient-derived rhabdomyosarcoma cell lines Rh18 (eRMS) and Rh30 (aRMS) lines were obtained from Dr. P. Houghton (St. Jude Children’s Hospital, Memphis, TN). Stable Rh18 and Rh30 cell lines were generated using the SparQ Cumate Switch Inducible System (System Biosciences, CA) as described previously ^12^. All RMS cell lines were maintained in RPMI-1640 medium supplemented with 10% FBS and 1% penicillin/streptomycin while the stable RMS cell lines were supplemented additionally with 100 µg/mL geneticin (Life Technologies, CA), and 2 µg/mL puromycin (Corning, NY). Primary human skeletal muscle myoblasts (HSMM) (Lonza, Walkersville, MD) were maintained in Clonetics SkGM™ medium supplemented with the SkGM™ SingleQuots™ kit (Lonza) as specified by the supplier. The HSMM were differentiated by switching to DMEM medium with 2% horse serum ^6^. Quercetin was purchased from Sigma-Aldrich (St. Louis, MO) and prepared in DMSO. Quercetin was used at 50 μM and 10 μM for Rh18 and Rh30 cells, respectively, for 24 hours unless indicated otherwise. For experiments that required longer quercetin treatment than 24 hours, the drug was refreshed in culture medium every 24 hours. All transfections were performed with Lipofectamine 2000 Reagent (Thermo Scientific, Waltham, MA). All cell cultures were incubated at 37°C, 5% CO_2_ and tested negative for mycoplasma.

## Western Blotting

Cells were lysed in IP lysis buffer (150 mM NaCl, 10 mM Tris-HCl, pH 7.4, 1 mM EDTA, 0.5% NP-40, and 1% Triton X-100) as previously described ^30^. The lysates were separated in 10% SDS-PAGE and transferred to PVDF membranes. The proteins were immunoblotted with anti-PANX1 (1:2000, Sigma-Aldrich, Cat#: HPA016930), anti-MyoD (1:1000, Santa Cruz, TX, Cat#: sc-377460), anti-MYOG (1:1000, Santa Cruz, Cat#: sc-12732), anti-MHC (1:2000, R&D Systems, Minneapolis, MN, Cat#: MAB4470), anti-CREB (1:1000, Cell Signaling Technology, Danvers, MA, Cat#: 4820S), anti-pCREB (1:1000, Cell Signaling Technology, Cat#: 9198S), anti-ETV4 (1:500, Santa Cruz, Cat#: sc-166629), or anti-GAPDH (1:5000, Advanced ImmunoChemical, Long Beach, CA, Cat#: 2RGM2) and then with Alexa 680- (1:5000, Thermo Scientific, Cat#: A-31553) or infrared fluorescent-labeled secondary antibodies IRDye 800 (1:5000, Rockland Immunochemicals, Pottstown, PA, Cat#: 610-132-121). The membranes were scanned on a LI-COR infrared-imaging system (LI-COR Biosciences, Lincoln, NE) and the densitometric units of the unsaturated images was quantified with the accompanying Odyssey V3.0 software.

## RNA Interference

Cells were transfected with 5 nM of Silencer® Select siRNA targeting *PANX1* (Sense: CGAUCAGUUUCAGUGCAAAtt; anti-sense: UUUGCACUGAAACUGAUCGgg) or Silencer® Select Negative Control No. 1 (Life Technologies) for 72 hours before quercetin treatment or Western blotting analysis.

## RNA Sequencing and Data Analysis

Total RNA was extracted from Rh30 cells using RNeasy Mini Kit (Qiagen, Germantown, MD) and submitted to Princess Margaret Genomics Centre (Toronto, ON, Canada) for RNA-seq analysis on an Illumina HiSeq2000 sequencing platform as previously described (Xiang et al., 2020). The RNA Integrity Number (RIN) was 10 for all sequenced samples. Total purity filtered reads sequenced generated were 80,991,098, 93,328,541, and 156,294,622 for the biological replicates. Overall read quality was checked using FASTQC v.0.11.2 RNA-SeQC (v1.1.7) software. Raw sequence data, in the form of FASTQ files, was aligned to the human genome (hg19, iGenome GTF definition file) using the BOWTIE/TOPHAT pipeline (BOWTIE v2.2.3, TOPHAT 2.0.13). Accessory programs for the alignment stage included SAMTOOLS (v1.0) and CUTADAPT (v1.7.1).

## Polysome profiling

Polysome profiling was performed as previously described (Gandin et al., 2014). Briefly, cells were treated with cycloheximide (CHX) (Bioshop, Canada) at 100 µg/ml for 5 minutes to fix translating ribosome. Cells were washed three times with ice cold PBS containing CHX, then lysed using hypotonic buffer (5 mM Tris pH 7.5, 2.5 mM MgCl_2_, 1.5 mM KCl, 2.5 mM DTT, 100U RNAsin nuclease inhibitor (Promega), 0.5% Triton X-100, 0.5% sodium deoxycholate). Cell debris was pelleted by centrifugation at 17,000 g, 10 minutes at 4^o^C and supernatant was transferred to a new tube. An equivalent of 10 OD at 260 nm of supernatant was then added on top of a continuous 10-50% sucrose gradient and centrifuged at 221,000 g (36,000 rpm) for 2 hours at 4^o^C. Fractions were then collected using a BR-188 Density Gradient Fractionation System (Brandel) and RNA was extracted using Trizol (Thermo Fisher) according to the manufacturer protocol.

## RT-qPCR

Total RNA was extracted using RNeasy Mini kit (Qiagen, Germantown, MD), DNase-treated with TURBO DNA-*free*^TM^ kit (Thermo Scientific) and reverse transcribed into cDNA using High-Capacity cDNA Reverse Transcription kit (Thermo Scientific). Quantitative PCR of the synthesized cDNA was performed on a CFX96 Touch Real-Time PCR Detection System (Bio-Rad, Hercules, CA) using SsoAdvanced^TM^ Universal SYBR^®^ Green Supermix (Bio-Rad). All primers were validated to have an amplification efficiency of 90 – 100% when used at 500 nM (see Supplemental Table S1 for primer sequences) with their respective qPCR program. All qPCR assays were performed using the following program unless otherwise specified: 95 ℃ for 3 min followed by 45 cycles of 95 ℃ for 10 sec, 58 ℃ for 15 sec and 72 ℃ for 50 sec). Relative expression was determined using the comparative Ct method.

## 5’ Rapid Amplification of cDNA End (RACE)

Rh30 (aRMS) cells were treated with 10 µM quercetin or DMSO for 24 hours, while HSMM were used at 0 (undifferentiated) or 48 hours (differentiated) following serum starvation to induce differentiation. Total RNA was collected and DNase-treated as described previously. Reverse transcription of 5’ capped poly (A) RNA was performed with TELO^TM^ PRIME Full-Length cDNA Amplification V2 kit following manufacturer’s instructions. Briefly, 2 µg of DNase-treated total RNA was first reverse transcribed using an OligodT primer to form a cDNA/RNA hybrid strand. A double-stranded adapter with a 5’ C overhang was then ligated to the 5’ end of the capped cDNA/RNA by base pairing to the 5’ G in the cap structure while leaving all 5’ uncapped cDNA/RNA unmodified. Full double-strand cDNA was then synthesized from the 5’ capped RNA using a primer containing the 5’ adapter sequence. Endpoint PCR using a forward primer with the 5’ adaptor sequence and a reverse primer specific to exon 2 of *PANX1* (Supplemental Table S1) were used to amplified full length *PANX1* cDNA for 21 PCR cycles without allowing the amplification to reach saturation (determined by a separate qPCR analysis under identical conditions). The PCR products were resolved on a 2% agarose gel with GelRed^TM^ (Biotium, Fremont, CA) and visualized on a Gel Doc XR+ Documentation System (Bio-Rad). All PCR programs were set up according to the manufacturer’s instructions.

## CAT Translation Reporter Assay

CAT translation reporter assay was performed as previously described (Graber et al., 2010). Briefly, cells were seeded at 75% confluency in a 6-well plate, then transfected with 1 µg each of pBGal (expressing β-galactosidase, a generous gift from Dr. Martin Holcik at Carleton University) and the pMCpA plasmid expressing the CAT open reading frame under the CMV promoter followed by the 5’UTR of interest using Lipofectamine 2000 (Thermo Fisher). 24 hours post transfection, CAT expression in transfected cells was measured using the CAT ELISA kit (Roche) according to the manufacturer’s instruction, while β-galactosidase activity was measured by the ortho-Nitrophenyl-β-galactoside (ONPG) colorimetric assay. CAT expression was then normalized to β-galactosidase to control for transfection efficiency.

## PANX1 Promoter Cloning, Plasmid Construction and Site-directed Mutagenesis

Total genomic DNA was extracted from primary HSMM cells using QIAamp DNA Mini kit (Qiagen). Two segments of *PANX1* promoter from positions -2697 to -1642 and -1616 to +38 as well as their deletion mutants from positions -926, -581 and -475 to +38 (relative to its ATG start codon) were PCR amplified (95 ℃ for 3 min followed by 32 cycles of 95 ℃ for 15 sec, 60 ℃ for 15 sec and 72 ℃ for 90) using PCR Master Mix (Promega, Madison, WI), purified with QIAquick PCR Purification kit (Qiagen), and subcloned into the KpnI-XhoI sites in pGL3-Basic vector (Promega). The lack of the 26 bp between -1642 and -1616 in the *PANX1* promoter is due to challenges in cloning this GC-rich region. *In silico* prediction yielded no potential transcription factors in this short fragment.

Mutations at the CREB and ETV4 consensus sites on *PANX1* promoter (-581 to +38) in pGL3 vector were introduced using the Quick-Change Site-directed Mutagenesis kit (Agilent Technologies, Santa Clara, CA) following the manufacturer’s instructions. All plasmid constructs were verified by Sanger sequencing. All restriction enzymes were purchased from New England Biolabs (Ipswich, MA). See Supplemental Table S1 for primer sequences.

## Immunofluorescence Microscopy

Rh30 cells on glass coverslips were treated with 10 µM quercetin or DMSO for 24 hours and then fixed with 3.7% paraformaldehyde for 20 min at room temperature. The fixed cells were washed with PBS, and then blocked and permeabilized in 2% BSA with 0.1% Triton X-100 for 1 hour at room temperature. The cells were labeled with previously described primary antibodies against PANX1 (1:200, Sigma-Aldrich, Cat#: HPA016930) and ETV4 (1:50, Santa Cruz, Cat#: sc-166629) at 4 ℃ overnight and then with Alexa 488 (Thermo Fisher, Cat#: A-11001) or 594 (Thermo Fisher, Cat#: A-11012) conjugated secondary antibodies (1:500) at room temperature for 1 hour. The coverslips were mounted with DAPI Fluoromount-G (Southern Biotech, Birmingham, AL) and visualized using EVOS Cell Imaging System (Thermo Fisher) with a 20X objective.

## Dual-luciferase Reporter Assay

Rh18 (eRMS) and Rh30 (aRMS) cells were transfected in 96-well plates with 100 ng pGL3-*PANX1* promoter constructs or a promoterless pGL3-Basic negative and 100 ng pRL-TK (Promega) for 48 hours and analyzed using Dual-Glo® Luciferase Assay System (Promega) on a Synergy HTX plate reader (BioTek Instruments, Winooski, VT). Rh18 and Rh30 cells were also transfected in parallel with 1 µg pGL3 constructs and 1 µg pRL-TK in 6-well plates for 48 hours before subjected to total RNA extraction for qPCR analyses of *Firefly Luciferase* and *PANX1* 5’ UTR transcript levels (95 ℃ for 3 min followed by 45 cycles of 95 ℃ for 3 sec and 60 ℃ for 30 sec) with primers described in Supplemental Table S1.

## Chromatin Immunoprecipitation

Rh30 (aRMS) cells were treated with 10 µM quercetin or DMSO for 24 hours and subjected to chromatin immunoprecipitation using EZ-ChIP kit (Millipore, Billerica, MA) according to manufacturer’s instructions. Briefly, the cells were fixed with 1% formaldehyde for 10 minutes at room temperature and quenched with 125 mM glycine. The cells were pelleted, and then lysed at 1 × 10^7^ cells/mL of SDS lysis buffer (1% SDS, 10 mM EDTA, and 50 mM Tris, pH 8.1) containing protease inhibitors. The cell lysates were sonicated by a VCX 130 sonicator (Sonics & Materials, Newtown, CT) mounted with a 2 mm tip for 15 sets of 10 s pulses at 30% of maximum power (50 Watts) to shear the crosslinked chromatin into 200 – 1000 bp fragments. Each immunoprecipitation was performed with 100 µL of sonicated chromatin, which was equivalent to 1 × 10^6^ cells. The chromatin was diluted to 1 mL with the dilution buffer and precleared with 60 µL of protein G agarose beads for 1 hour at 4 ℃. A 20 µL aliquot of precleared chromatin was removed and set aside to be used as input in subsequent analysis. The precleared chromatin was then incubated with 1 µg anti-RNA Polymerase II (positive control), 1 µg mouse IgG (negative control), 10 µg anti-ETV4 (Santa Cruz; Cat#: sc-166629X) or 10 µg anti-CREB (Santa Cruz; Cat#: sc-377154X) overnight at 4 ℃ on a rotator. The chromatin/DNA immunocomplexes were captured with 60 µL of protein G agarose beads for 1 hour at 4 ℃. The protein G agarose beads were washed, and the captured chromatin was eluted and purified for subsequent qPCR analysis (95 ℃ for 3 min followed by 45 cycles of 95 ℃ for 3 sec and 60 ℃ for 30 sec) using primers described in Supplemental Table S1.

***3D Spheroid Assay***

3D spheroid formation and regression assays were performed and quantified using IncuCyte ZOOM^TM^ Live Cell Imaging System (Essen Bioscience, MI) and its accompanying software as previously described ^12^

## Statistics

Paired or unpaired two-tailed Student’s *t*-tests, multiple Student’s *t*-test with Holm-Sidak correction, and one-way or two-way analysis of variance (ANOVA) followed by Tukey’s *post hoc* tests were used. Results are given as mean ± s.d. Results with *P* < 0.05 were considered significant. The exact number of times each experiment was performed is indicated in the Figure legends with the individual data points displayed on the graphs.
