# Supplementary material for: Quercetin induces pannexin 1 expression via an alternative transcript with a translationally active 5′ leader in rhabdomyosarcoma
Source: Oncogenesis. 2022 Feb 22;11(1):9. doi: 10.1038/s41389-022-00384-9 (PMC8864035; doi:10.1038/s41389-022-00384-9)
Supplement: Supplementary file 2 — Supplementary Information (Table S1 - List of Primers) [file 41389_2022_384_MOESM2_ESM.docx]

# SUPPLEMENTARY INFORMATION

**Table S1. List of Primers**

| **Assay** | **Primer Name** | **Sequence (5' to 3')** |  |
| --- | --- | --- | --- |
|  |  |  |  |
| PANX1 Promoter Cloning | -1616PANX1pmt-Fwd | CCCAAGGAGCCCTCATGTTT |  |
|  | -926PANX1pmt-Fwd | AAGAGAAACGGGACCAGCAG |  |
|  | -581PANX1pmt-Fwd | CGCTCAGTGGGAAAATCCCT |  |
|  | -475PANX1pmt-Fwd | TGGGTACTTGGTTTCCCCG |  |
|  | +38PANX1pmt-Rev | GAGAACACGTACTCCGTGGC |  |
|  | -2679PANX1pmt-Fwd | CCTACCCTCAAGAAGCCGAT |  |
|  | -1642PANX1pmt-Rev | CTTGGCTCCCTTCTCTGTGG |  |
|  |  |  |  |
| PANX1 5' UTR Cloning |  |  |  |
|  |  |  |  |
|  |  |  |  |
| qPCR | 5'UTRPANX1-Fwd | GGACTTGCACGGGCG |  |
|  | 5'UTRPANX1-Rev | GTACCAATCGAGATCTCCTG |  |
|  | PANX1Exon1/2-Fwd | CACGATGGTCACGTGCATTG |  |
|  | PANX1Exon1/2-Rev | GTACCAATCGAGATCTCCTG |  |
|  | PANX1Exon2/3-Fwd | GCTGTTCAGCAGAAGAACTCAC | * |
|  | PANX1Exon2/3-Rev | TCTGAGCAAATATGAGGAGCAG | * |
|  | PANX1Exon4/5-Fwd | AAGTGTACGAAATCCTCCCC |  |
|  | PANX1Exon4/5-Rev | GTTCATACCTTGGAGCTCTGC |  |
|  | FireflyLuc-Fwd | CTCACTGAGACTACATCAGC |  |
|  | FireflyLuc-Rev | TCCAGATCCACAACCTTCGC |  |
|  | RenillaLuc-Fwd | GGAATTATAATGCTTATCTACGTGC |  |
|  | RenillaLuc-Rev | CTTGCGAAAAATGAAGACCTTTTAC |  |
|  | 5'UTRPANX1(-309)-Fwd | GAGGCGCGAATCCGAGTG |  |
|  | 5'UTRPANX1(-309)-Rev | CGGGCCTTTCTTTATGTTTTTGG |  |
|  | 5'UTRPANX1(-257)-Fwd | GGGTGGAACCGCAGGAAG |  |
|  | 5'UTRPANX1(-257)-Rev | GCCGGGCCTTTCTTTATGTTTTT |  |
|  | 5'UTRPANX1(-156)-Fwd | CTGAGGCACCGAGACACAAA |  |
|  | 5'UTRPANX1(-156)-Rev | GCCGGGCCTTTCTTTATGTTTTT |  |
|  | 5'UTRPANX1(-113)-Fwd | CAAAGGGAAAGCGAAAGCCG |  |
|  | 5'UTRPANX1(-113)-Rev | GCCGGGCCTTTCTTTATGTTTTT |  |
|  | 5'UTRPANX1()-Fwd | CCATGGCCATCGCTCAACT |  |
|  | 5'UTRPANX1()-Rev | GCCGGGCCTTTCTTTATGTTTTT |  |
|  | GAPDH-Fwd | GTCTCCTCTGACTTCAACAGCG |  |
|  | GAPDH-Rev | ACCACCCTGTTGCTGTAGCCAA |  |
|  |  |  |  |
| Mutagenesis*** | mutCREB-Fwd | CGCCCACCCCGC**CCCTTACCA**CCGCGTCTTCCGG | |
|  | mutCREB-Rev | CCGGAAGACGCGG**TGGTAAGGG**GCGGGGTGGGCG | |
|  | mutETV4-Fwd | CCCGCGTCACCGCG**TCCCAAGG**AAGCTCCACGC |  |
|  | mutETV4-Rev | GCGTGGAGC**TTCCTTGGGA**CGCGGTGACGCGGG |  |
|  | mutCREBmutETV4-Fwd | GC**CCCTTACCA**CCGCG**TCCCAAGG**AAGCTCCA |  |
|  | mutCREBmutETV4-Rev | TGGAGCTT**CCTTGGGA**CGCGG**TGGTAAGGG**GC |  |
|  |  |  |  |
| ChIP | CREB-Fwd | CGCTCAGTGGGAAAATCCCT |  |
|  | CREB-Rev | GGGGAAACCAAGTACCCAGG |  |
|  | ETV4-Fwd | GCCAGGACGTGAGGAGAATC |  |
|  | ETV4-Rev | CCATGCGGGGAAACCAAGTA |  |
|  | GAPDH_ChIP-Fwd | TACTAGCGGTTTTACGGGCG |  |
|  | GAPDH_ChIP-Rev | TCGAACAGGAGGAGCAGAGAGCGA |  |
|  |  |  |  |
| 5' RACE | TeloFwd | TGGATTGATATGTAATACGACTCACTATAG | ** |
|  | TeloRev | TCTCAGGCGTTTTTTTTTTTTTTTTTT | ** |
|  | PANX1Exon2-Rev | GGGAGGTTTCCAGACTCGC |  |
|  |  |  |  |
|  |  |  |  |
| *Validated primer set purchased from Genecopoeia | |  |  |
| **Primer sets provided in the TeloPrime Full-Length cDNA Amplification Kit V2 | | |  |
| ***Concensus sites are in bold and mutated bases are underlined | | |  |
